# Sex- and Regio-Specific Lipid Profiling of Shishamo and Capelin Fish by Nontargeted Liquid Chromatography/Mass Spectrometry

**DOI:** 10.3390/foods15020298

**Published:** 2026-01-14

**Authors:** Yusuke Minami, Siddabasave Gowda B. Gowda, Divyavani Gowda, Hitoshi Chiba, Shu-Ping Hui

**Affiliations:** 1 Graduate School of Health Sciences, Hokkaido University, Kita-12, Nishi-5, Kita-ku, Sapporo 060-0812, Japan; minami.yusuke.t2@elms.hokudai.ac.jp; 2 Faculty of Health Sciences, Hokkaido University, Kita-12, Nishi-5, Kita-ku, Sapporo 060-0812, Japan; divyavani@hs.hokudai.ac.jp; 3 Graduate School of Global Food Resources, Hokkaido University, Kita-9, Nishi-9, Kita-Ku, Sapporo 060-0809, Japan; 4 Department of Nutrition, Sapporo University of Health Sciences, Nakanuma, Nishi-4-3-1-15, Higashi-ku, Sapporo 007-0894, Japan; chiba-h@sapporo-hokeniryou-u.ac.jp

**Keywords:** liquid chromatography, fish lipids, mass spectrometry, lipidomics, fatty acid, shishamo

## Abstract

Shishamo smelt (*Spirinchus lanceolatus*), which is endemic to Hokkaido, Japan, is frequently substituted in markets with morphologically similar capelin (*Mallotus villosus*) imported from abroad. Lipids are essential nutrients that play important roles in fish authenticity, validation, and nutritional assessment. Although shishamo has long been consumed in Japan, its region-specific lipid distribution and composition are different from those of capelin and have not been well explored. To overcome these gaps, we used untargeted liquid chromatography–mass spectrometry to profile sex- and region-specific lipids in the Japanese *S. lanceolatus* and Nordic *M. villosus*. The results revealed that female *S. lanceolatus* heads exhibited high triacylglycerol (TAG) content, closely resembling roe composition. Docosahexaenoic acid and eicosapentaenoic acid were enriched in the female *S. lanceolatus* viscera. Multivariate analysis identified monounsaturated fatty acids, such as fatty acid (FA) 22:1 and FA 20:1, as robust discriminatory markers between *S. lanceolatus* and *M. villosus*. Overall, sex- and regiospecific differences in lipid composition between the two species were correlated and compared. These lipidomic signatures provide a basis for verifying species authenticity and geographic origin, while highlighting the nutritional lipid potential of *S. lanceolatus*, particularly from the female viscera.

## 1. Introduction

Shishamo smelt (*Spirinchus lanceolatus*) is an endemic species restricted to Hokkaido, the northernmost island of Japan [1]. In early winter, it has an anadromous life cycle, migrating from the coastal ocean to the lower reaches of rivers to spawn [2]. Owing to its restricted distribution and seasonally limited landings, shishamo is a culturally and economically important fishery resource in Hokkaido. However, shishamo populations have declined markedly, leading to its classification as an endangered species on Japan’s Red List [1,3]. Capelin (*Mallotus villosus*), a closely related osmerid often referred to in Japan as “karafuto-shishamo,” occupies sub-Arctic pelagic habitats throughout the Northern Hemisphere [4]. Although a large proportion of the global capelin catch is processed into fishmeal, prespawning individuals are also valued for direct human consumption because of their favorable nutritional profiles [5]. In Japan, the majority of “shishamo” marketed is imported frozen capelin, yet the biological and nutritional differences between shishamo and capelin are poorly understood [6].

Fish lipids are a well-recognized dietary source of bioactive compounds with significant health benefits [7]. These benefits are largely attributed to their high content of ω-3 polyunsaturated fatty acids (PUFAs), particularly eicosapentaenoic acid (EPA) and docosahexaenoic acid (DHA) [8]. ω-3 PUFAs lower circulating triacylglycerol levels and reduce the risk of neurodegenerative disorders, including Alzheimer’s disease, dementia, and certain cancers [9]. By contrast, saturated fatty acids (SFAs), especially long-chain SFAs (LCSFAs), are associated with elevated serum cholesterol and increased cardiovascular risk, as suggested by previous studies [10]. To evaluate the nutritional quality of fish-derived lipids, free fatty acid (FFA)-based indices such as the PUFA/SFA ratio, index of atherogenicity (IA), and hypocholesterolemic/hypercholesterolemic (HH) ratio, are widely employed [11].

Advances in lipidomics now allow for the comprehensive characterization of food lipidomes and nutritional profiles beyond FFA-based assessments [12]. Lipid composition in fish is modulated by species, age, diet, and environment [13]. Lipidomics provides valuable insights into species-specific nutritional properties, feeding ecology, and even geographic origin [14]. For instance, in capelin (*Mallotus villosus*), ω-3 PUFAs account for ~71% of total lipids, with EPA and DHA constituting over 16% [5]. Long-chain PUFAs in food typically occur in triacylglycerols (TAGs) and phospholipids (PLs) [15], and evidence suggests that ω-3 PUFAs in TAG and PL forms enhance tissue incorporation more effectively than free forms [16]. Therefore, comprehensive lipidomic analyses, including TAGs, PLs, and other complex lipid classes, are crucial for evaluating the nutritional and functional potential.

Sex-and region-specific differences also influence fish lipid profiles. For example, ayu (*Plecoglossus altivelis*) roe contains nearly three times more phospholipids than muscle [17], and female capelin accumulates greater lipid reserves in the gonads than males [18]. Recently, organ-specific lipidomic maps of capelin revealed that ceramides (Cer) are enriched in the viscera and phosphatidylcholine (PC) is enriched in the roe [5]. Our previous work analyzing Japanese marine products demonstrated that female shishamo smelt (*Spirinchus lanceolatus*) meat is particularly rich in ω-3 PUFAs [19]. However, sex- and region-specific differences in lipid composition between shishamo and capelin have not yet been fully elucidated. In the current study, we performed a comprehensive lipidomic comparison of shishamo from Hokkaido, Japan, and capelin from Nordic countries, two species widely consumed in Japan but often confused in markets. Using liquid chromatography/mass spectrometry (LC/MS)-based untargeted lipidomics, we assessed the lipid composition across multiple body parts (head, meat, viscera, and roe) and sexes. This approach enabled us to identify species-, sex-, and region-specific lipid signatures, discuss their nutritional implications, and propose candidate molecular markers to distinguish shishamo from capelin.

## 2. Materials and Methods

### 2.1. Materials

LC-MS-grade solvents, including methanol, isopropanol, and chloroform, were purchased from Wako Pure Chemical Industries (Osaka, Japan). Ammonium acetate (1 M), used as the mobile phase additive, was obtained from Sigma-Aldrich (St. Louis, MO, USA). For sample homogenization, 1.4 mm ceramic beads (item no. 15-340-159) were purchased from Thermo Fisher Scientific (Tokyo, Japan). Pierce™ LTQ ESI Positive Ion Calibration Solution and Pierce™ Negative Ion Calibration Solution were obtained from Thermo Fisher Scientific (Tokyo, Japan). To prepare the internal standards, EquiSPLASH LIPIDOMIX and oleic acid (d9) were obtained from Avanti Polar Lipids, Inc. (Alabaster, AL, USA). A mixed internal standard solution was prepared in methanol (1 μg/mL) containing: phosphatidylcholine (PC 15:0/18:1(d7)), phosphatidylethanolamine (PE 15:0/18:1(d7)), phosphatidylglycerol (PG 15:0/18:1(d7)), phosphatidylserine (PS 15:0/18:1(d7)), phosphatidylinositol (PI 15:0/18:1(d7)), lysophosphatidylethanolamine (LPE 18:1(d7)), lysophosphatidylcholine (LPC 18:1(d7)), sphingomyelin (SM d18:1/18:0(d9)), ceramide (Cer d18:1/15:0(d7)), monoacylglycerol (MAG) (18:1(d7)), diacylglycerol (DAG), triacylglycerol (TAG) (15:0–18:1(d7)-15:0), and cholesterol ester (18:1(d7)) along with 10 μg/mL oleic acid (d9).

### 2.2. Sample Preparation

Male and female shishamo smelt (*Spirinchus lanceolatus*) from Hokkaido, Japan, were obtained from Nomoto Suisan Co., Ltd. (Hokkaido, Japan). Male capelin (*Mallotus villosus*) from Iceland was purchased from Taikai Co., Ltd. (Tottori, Japan), and female capelin from Norway was purchased from Kanenaka Food Industry Co., Ltd. (Ishikawa, Japan). Sex and geographical origin were completely confounded in the current study. This mixed design makes it difficult to disentangle the independent contributions of sex-specific effects (e.g., hormonal differences) and region-specific effects (e.g., feeding grounds or environmental factors), which may complicate the interpretation of their interactions. Head, meat, viscera, and roe (female only) were dissected within 10 min of arrival and stored at −80 °C until extraction. Four individuals of each fish species were prepared, dissected to obtain specific tissues, and subjected to four replicate analyses.

### 2.3. Lipid Extraction

Approximately 100 mg of tissue was homogenized with 1.4 mm ceramic beads (Beadmill 4, Fisher Scientific, Tokyo, Japan). One milliliter of methanol containing 0.01% butylated hydroxytoluene was added, followed by the extraction of total lipids using a modified Folch method [19,20]. Each homogenate was spiked with an internal standard, mixed with chloroform and Milli-Q water, and then centrifuged. The chloroform phase was collected, re-extracted, dried under vacuum, and reconstituted in 100 µL methanol. After centrifugation at 15,000 rpm (10 min, 4 °C), the supernatant was transferred to autosampler vials for LC/MS analysis.

### 2.4. LC/MS Analysis

For LC-MS analysis, a protocol similar to that reported in our previous study was followed [21]. Lipid profiling was performed using a Prominence UFLC system (Shimadzu, Kyoto, Japan) coupled to an LTQ Orbitrap mass spectrometer (Thermo Fisher Scientific, San Jose, CA, USA) with an Atlantis T3 C18 column (2.1 mm × 150 mm, 3 µm, Waters). The column temperature was maintained at 40 °C. Lipids were separated using (A) Milli-Q (10 mM ammonium acetate), (B) isopropanol, and (C) methanol with gradient elution in both positive and negative mode at 200 µL/min over 30 min. Eluent distribution in positive mode was 30% B and 35% C (0–1 min), 82.5% B and 15% C (1–9 min), 95% B and 5% C (9–15 min), 95% B and 5% C (15–25 min), 30% B and 35% C (25–26 min) and equilibrated for 4 min. Eluent distribution in negative mode was 30% B and 35% C (0–1 min), 75% B and 15% C (1–9 min), 82.5% B and 15% C (9–21 min), 95% B and 5% C (21–25 min), 30% B and 35% C (25–26 min), and equilibrated for 4 min. Data were acquired in electrospray ionization (ESI) positive and negative modes, with the following conditions: capillary temperature 330 °C; nitrogen sheath gas flow rate 50 units; auxiliary gas flow rate 20 units (positive) and 30 units (negative); source voltage 4 kV (positive) and 3 kV (negative); and capillary voltage 25 V (positive) and 10 V (negative). The MS^1^ scan ranges were *m*/*z* 100–1750 (positive), and *m*/*z* 160–1900 (negative) in Fourier transform mode with a resolution of 60,000. MS^2^ and MS^3^ spectra were acquired using collision-induced dissociation (CID) at collision energies of 40 and 45 eV, respectively. Calibration was performed using Pierce™ LTQ ESI Positive Ion Calibration Solution and Pierce™ Negative Ion Calibration Solution (both from Thermo Fisher Scientific, Tokyo, Japan). Before sample analysis, blanks were used to confirm the absence of contamination and quality control (QC) samples were used to monitor the performance of the analytical instrument.

### 2.5. Data Processing for Identification of Lipids

Alignment, lipid annotation, and peak area integration of the raw data acquired by LC-MS analysis were performed using MS-DIAL ver. 5.1. The parameters set in MS-DIAL were the same as previously reported [22], specifically employing a minimum peak height of 1000 amplitude units, 0.1 Da mass slice width, smoothing across 3–5 scans with a 0.5 sigma window, signals at least fivefold above blank levels, and tolerances of 0.5 min for retention time alongside 0.015 Da for MS^1^. To confirm the identification of lipid molecular species, MS^1^, MS^2^, and MS^3^ spectra were confirmed using Xcalibur 2.2 (Thermo Fisher Scientific, Waltham, MA, USA). Relative quantification was performed using internal standards of the same or analogous lipid subclasses. Analyte levels were determined by multiplying the peak area ratio (analyte/internal standard) by the internal standard concentration and normalized to sample weight, following the Level 2 or 3 guidelines of the Lipidomics Standard Initiative.

### 2.6. Determination of Lipid Nutritional Indices

For nutritional assessment based on FFA, the following formulas were used to calculate the nutritional indices: fish lipid quality (FLQ), hypo/hypercholesterolemic ratio (HH), and index of atherogenicity (IA) [11].

FLQ = 100 × (C20:5 + C22:6)/ΣFA

HH = (C18:1 + ΣPUFA)/(C12:0 + C14:0 + C16:0)

IA = [C12:0 + (4 × C14:0) + C16:0]/ΣUFA

### 2.7. Statistical Analysis

Multivariate analyses (principal component analysis, heatmaps, and orthogonal partial least squares discriminant analysis) were performed using MetaboAnalyst 6.0, and additional graphs/statistics were generated using GraphPad Prism 8. Data are expressed as mean ± standard error (SE). Group comparisons for head, meat, and viscera were assessed using one-way analysis of variance (ANOVA) with Tukey’s post hoc test; roe lipids were compared using Student’s *t*-test. Distinct letters in the figures denote significant group differences (*p* < 0.05).

## 3. Result & Discussion

### 3.1. Lipid Annotation and Distribution of Major Lipid Classes

**Figure 1:**
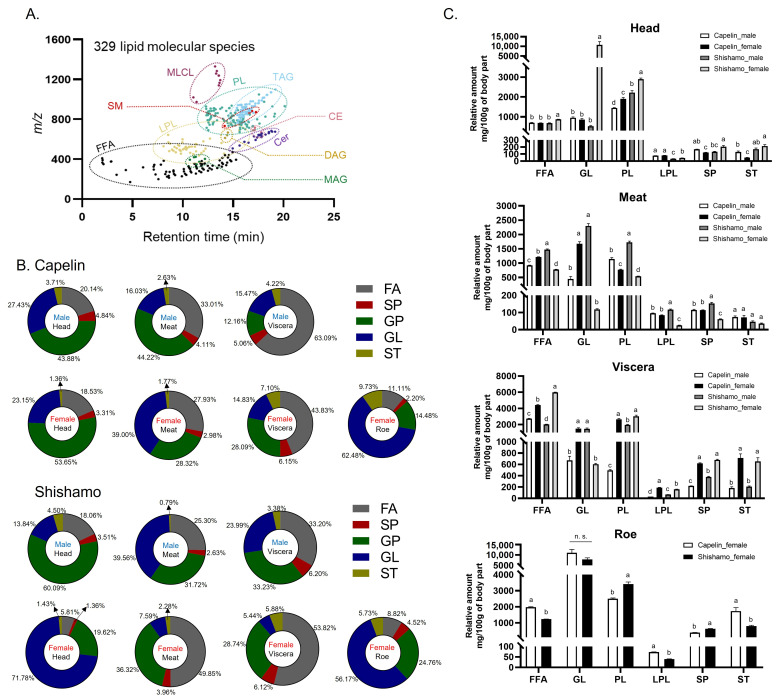
(**A**) *m*/*z* vs. retention time (RT) plot of detected lipids in shishamo and capelin. (**B**) Species, sex, body parts-specific percentage distribution of five lipid classes (FA: fatty acids, SP: sphingolipids, GP: glycerophospholipids, GL: glycerolipids, ST: sterol lipids). (**C**) Relative amount of six lipid classes based on body part (FFA: free fatty acids, PL: phospholipids, LPL: lyso-phospholipids). Different letters (a–d) indicate significant differences (*p* < 0.05); n.s. indicates no significant difference.

Untargeted LC-MS analysis annotated 329 lipid molecular species in *Spirinchus lanceolatus* (shishamo) and *Mallotus villosus* (capelin) in both positive and negative ionization modes (Supplementary Table S1). Scatter plots (*m*/*z* vs. retention time) representing the elution of different lipid subclasses are shown in Figure 1A. This illustrates the distribution of the detected lipid species, which were classified into five major categories: fatty acyls (FA), sphingolipids (SP), glycerophospholipids (GP), glycerolipids (GL), and sterols (ST). The relative abundances of these categories differed considerably according to the species, sex, and body region (Figure 1B). In capelin, GP predominated in male head (43.9%) and meat (44.2%), whereas FA dominated viscera (63.1%). Female capelins also exhibited high GP in the head (53.7%), high FA in viscera (43.8%), and high GL in meat (39.0%) and roe (62.5%). By contrast, male shishamo heads were GP-rich (60.1%), while meat contained elevated GL (39.6%), and viscera showed balanced GP and FA (~33% each), respectively. Elevated levels of GPs were detected in the lipid profile of the golden threadfin bream head [23]. Interestingly, female shishamo heads were GL-dominated (71.8%), a unique lipid feature absent in the other head samples. Previous studies have reported that TAG, a type of GL, is the predominant lipid class in salmon head paste [24]. Female shishamo meat (49.9%) and viscera (53.8%) were FA-rich, while roe was dominated by GL (56.2%). The relative abundance of lysophospholipids (LPL) along with other major lipid classes by body region (Figure 1C) further highlighted differences: female shishamo heads contained significantly higher GL and PL levels ranked as capelin males < capelin females < shishamo males < shishamo females, and male shishamo meat exhibited the highest levels across nearly all classes except ST. In the viscera, females of both species had significantly elevated FA, LPL, SP, and ST compared with males, and the storage and distribution of lipids were influenced by sex-related differences in energy allocation to reproduction and timing of migration [25]. The roe was uniformly dominated by GL, with no major interspecific differences.

### 3.2. Region-Specific Free Fatty Acid Profiles

**Figure 2:**
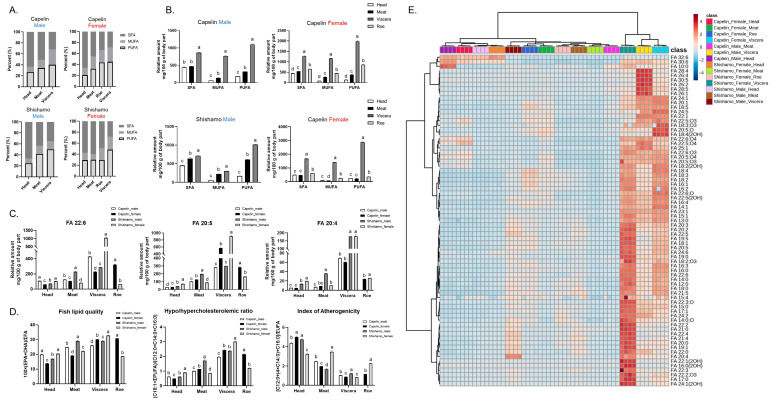
Free fatty acids (FFA) profiling in shishamo and capelin. (**A**) Percentage distribution of FFA based on unsaturation. (**B**) Relative amount of SFA, MUFA, PUFA. (**C**) Relative amount of FA 22:6, FA 20:5, and FA 20:4. (**D**) Lipid nutritional assessment by FFA (Fish lipid quality, Hypo/hypercholesterolemic ratio, index of atherogenicity). (**E**) Hierarchical Clustering Heat Map of free fatty acid, Data were quantile normalized followed by autoscaling for features (i.e., z-scores for each feature) using MetaboAnalyst 6.0. Different letters (a–d) indicate significant differences (*p* < 0.05).

Free fatty acids (FFAs) are key regulators of energy metabolism, gene expression, and signaling [26]. Tissue FFA composition was classified into saturated fatty acids (SFA), monounsaturated fatty acids (MUFA), and polyunsaturated fatty acids (PUFA) (Figure 2A). SFAs predominated in head tissues (57.6–66.5%), particularly in female shishamo meat (59.9%). By contrast, PUFAs were enriched in viscera across all groups (40.2–50.0%), and this trend largely reflects lipid storage in the liver and intestine, the major visceral organs. Semi-quantitative analysis confirmed that visceral FFA levels were substantially higher in females than in male-most notably, visceral PUFA reached 2873 ± 30 mg/100 g in female shishamo and 1981 ± 29 mg/100 g in female capelin (Figure 2B).

Among nutritionally relevant long-chain PUFAs (FA 22:6, FA 20:5, FA 20:4; Figure 2C), DHA (FA 22:6) was highest in female shishamo viscera (1048 ± 7 mg/100 g), followed by male capelin viscera (426 ± 7 mg/100 g). EPA (FA 20:5) was likewise elevated in shishamo female viscera (904 ± 15 mg/100 g) compared with capelin females (568 ± 8 mg/100 g). Arachidonic acid (FA 20:4; ω-6) showed no significant sex differences in shishamo, with similar levels in males and females (~179 mg/100 g). DHA and EPA are ω-3 PUFAs with well-documented cardioprotective and anti-inflammatory effects, whereas arachidonic acid (ω-6 PUFA) contributes to pro-inflammatory signaling, particularly when the ω-6/ω-3 ratio is elevated [27,28]. The high ω-3 PUFA content of both shishamo and capelin viscera suggests that these tissues may represent valuable dietary sources of health-promoting lipids. Chinese capelin roe contains higher levels of FA 22:6 and FA 20:5 than the viscera [5], and this difference may be related to the maturity of roe [29].

To assess the potential health effects of shishamo and capelin, lipid-based nutritional indices were calculated (Figure 2D). The fish lipid quality index (FLQ), which reflects the proportion of EPA and DHA relative to total FFA, is a widely used indicator of the nutritional value of fish lipids. FLQ values ranged from 13.8 to 32.7, consistent with previous reports for fish (13.0–36.4) [11]. The highest FLQ was observed in shishamo female viscera (32.7 ± 0.2), followed by capelin female roe (30.6 ± 0.2), while capelin female head yielded the lowest value (13.8 ± 0.2). The hypocholesterolemic/hypercholesterolemic ratio (HH), which predicts the effect of FA composition on serum cholesterol, was greatest in shishamo female viscera (3.01 ± 0.03), with lower values in capelin female viscera (2.43 ± 0.04) and shishamo male viscera (2.37 ± 0.04). Among roe, capelin exhibited a significantly higher HH ratio (2.15 ± 0.02) than shishamo (1.21 ± 0.01). These values align with literature ranges, where fish generally display ratios of 1.5–4.8, higher than meat (1.3–2.8) and dairy products (0.3–1.3) [11], supporting the relative nutritional advantage of fish lipids.

The index of atherogenicity (IA), used to evaluate the potential risk of lipid intake promoting atherosclerosis [30], was highest in capelin female head (5.0 ± 0.1) and shishamo male head (4.7 ± 0.1). Female shishamo meat also exhibited elevated IA (3.5 ± 0.1). By contrast, viscera across all groups showed comparatively low IA values (0.8–1.2), suggesting a reduced atherogenic risk from these tissues. Reported IA ranges were 0.2–1.4 for fish, 1.4–5.1 for dairy, and 0.2–1.3 for meat [11], indicating variability across food groups but underscoring the favorable profile of visceral lipids. All indices were calculated based on the amount of free fatty acids using formulas reported in our earlier studies [19,21]. Hierarchical clustering heatmaps (Figure 2E) highlighted the distinct differences in the FFA profiles between shishamo and capelin. Viscera consistently exhibited a high relative FFA abundance, particularly in females, except for shishamo males. In the heatmap, although samples from the same body part cluster together and the differences among body parts are much greater than those among species, these trends are primarily driven by fatty acid composition rather than the overall lipid composition. Moreover, our objective is to identify region- and sex-specific lipid profiles, which do not necessarily reflect drastic compositional differences. Lipid reserves serve as critical energy resources during gonadal development and sexual maturation and are known to vary with sex and reproductive stage [31]. Notably, very long-chain fatty acids (VLC-FAs; >C28), such as FA 32:6 and FA 30:6, were detected at relatively high levels in head tissues. The high VLC-FA content in head tissues may reflect contributions from specialized tissues, such as the retina [32,33], although specific functional attribution is difficult due to the use of composite head tissues. The identification of region-specific and sex-dependent FFA distribution in shishamo and capelin provides a valuable reference for future studies on fish nutrition, reproductive physiology, and biochemical adaptations.

### 3.3. Principal Component Analysis of Shishamo and Capelin by Sex

**Figure 3:**
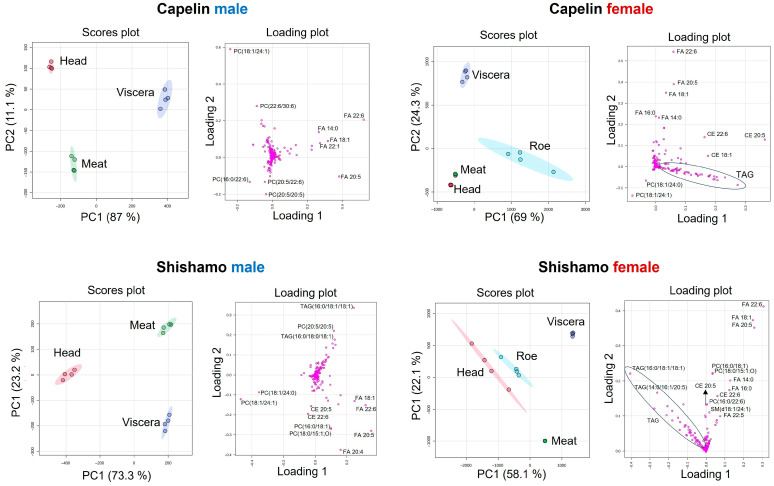
Principal component analysis (PCA) by species and sex (Score plot and Loading plot). Colored circles display 95% confidence regions.

Principal component analysis (PCA) was used to explore sex- and region-specific lipidomic variations in shishamo and capelin (Figure 3). The score plots demonstrated clear clustering by body part across all samples, reflecting a distinct lipid composition. The corresponding loading plots identified the lipid molecular species that contributed the most strongly to these separations. In male capelin, phosphatidylcholine (PC) species containing very long-chain FAs (e.g., PC(18:1/24:1), PC(30:6/22:6)) differentiated the head tissues, whereas omega-3-rich PCs (PC(16:0/22:6), PC(20:5/20:5), PC(20:5/22:6)) differentiated the meat. FFAs (FA 22:6, FA 20:5, FA 18:1) were the primary contributors to the viscera. Notably, VLC-FA-containing PCs are enriched in the head. This lipid is retinal-specific and supports normal visual function in mammals [34]. In female capelins, the head and meat profiles were more similar, with PCs (PC(18:1/24:0), PC(18:1/24:1)) as key discriminators. FFAs (FA 22:6, FA 20:5, FA 18:1) distinguish the viscera, whereas triacylglycerols (TAGs) and cholesterol esters (CE 22:6, CE 20:5, CE 18:1) are the defining features of roe. The enrichment of PC species in the head and meat is consistent with previous observations in other marine fish, such as saury [35]. Lipid profiling of Chinese female capelins revealed PC as a characteristic of roes [5], suggesting that this difference may be due to their geographic origin [36]. In male shishamo, heads were separated by PC species containing 24-carbon FAs (PC(18:1/24:0), PC(18:1/24:1)), whereas meat was differentiated by TAGs (TAG(16:0/18:1/18:1), TAG(16:0/18:0/18:1)), and PC(20:5/20:5). Viscera were characterized by FFAs (FA 22:6, FA 20:5, FA 18:1), with FA 20:4 showing strong negative loading on PC1. In female shishamo, PCA revealed a close association between the head and roe profiles, with the TAG species driving separation. This observation suggests the metabolic mobilization of TAGs from somatic tissues (e.g., muscle and viscera) into developing oocytes, as reported for other marine fishes [37,38]. The accumulation in the cranial tissues may represent an energy reserve that indirectly contributes to oogenesis. Furthermore, TAG distribution patterns in fish vary by order, species, and swimming mode [39]. Collectively, these PCA findings highlight species-, sex-, and region-specific lipidomic partitioning, reinforcing the distinctive head–roe link in female shishamo and the enrichment of VLC-FA-containing PCs in the capelin head tissue. Although lipidomic maps of capelin have recently been developed, this study provides, for the first time, a comprehensive region-resolved lipidomic profile of shishamo smelt.

### 3.4. Lipid Markers Distinguishing Shishamo from Capelin

**Figure 4:**
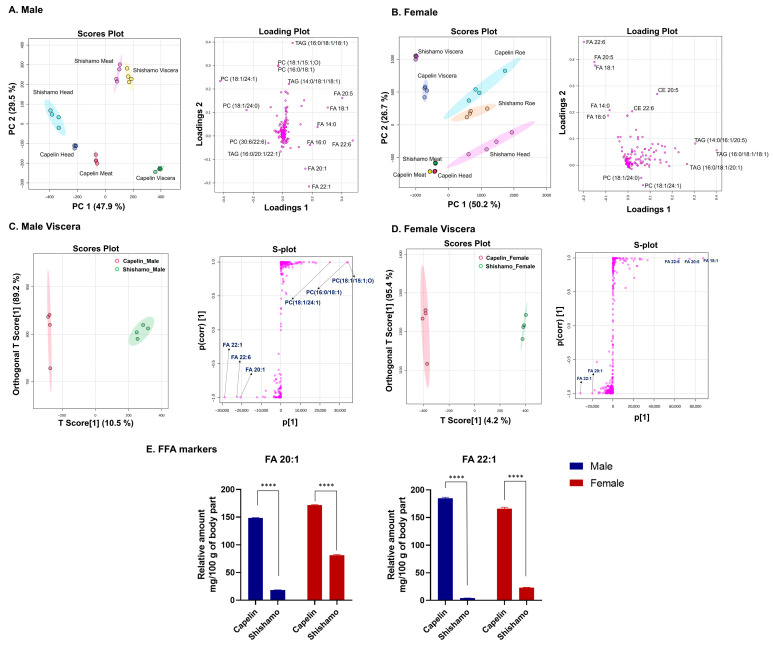
Multi-dimensional assessment of tissue-specific lipid markers for shishamo–capelin discrimination. (**A**) PCA for male, (**B**) PCA for female, (**C**) Orthogonal partial least squares discriminant analysis (OPLS-DA) for male, (**D**) OPLS-DA for fema*l*e, (**E**) Semi-quantitative levels (means ± SE, *n* = 4) of FA 20:1 and FA 22:1. **** *p* < 0.0001, Student’s *t*-test.

To identify the anatomical regions most suitable for the exploration of lipid-based species markers, Figure 4A,B depict PCA score plots for the head, meat, viscera, and roe (females only), comparing male shishamo vs. capelin and female shishamo vs. capelin. The PCA score plots for males revealed a distinct separation of muscle and viscera between shishamo and capelin. Loading plot analysis revealed that FFAs were the primary contributors to species separation in the viscera, whereas PC and TAG played key roles in the meat. In the female PCA score plots, the head and viscera were separated between species. Loading plot analysis revealed that in females, TAG contributed to separation in shishamo head tissue, whereas PC was the primary contributor to the capelin head. By contrast, FFA drives visceral separation in both species. The viscera facilitates marker discovery owing to the sole contribution of FFAs, while also enabling simplified analysis for practical applications. By contrast, in male meat, multiple factors, including PC and TAG, make marker identification challenging and complicate practical analysis. Orthogonal partial least squares discriminant analysis (OPLS-DA) of male meat tissues (Figure S1) further confirmed the multifactor complexity of the FA-TAG reversal patterns. Although the head tissue initially appeared promising, OPLS-DA (Figure S1) showed TAG reversal, which was abundant in shishamo female heads yet equally enriched in capelin female meat. This may reflect reproductive status [40], but the uncontrolled reproductive status in this study limits interpretation and deems the head unsuitable for species identification marker exploration. Furthermore, comparison of the roes (Figure S1), multiple lipid molecular species were intermixed, rendering interpretation challenging and imposing a high analytical burden for practical applications. These findings suggest that the viscera are an optimal tissue for lipid-based species identification.

For species discrimination, OPLS-DA analyses targeting visceral tissues were conducted separately for males and females (Figure 4C,D). Score plots showed a visual separation between shishamo and capelin for both sexes, with model robustness validated by 100 permutation tests confirming lipid composition differences (males: R^2^Y = 0.992, Q^2^ = 0.985; females: R^2^Y = 0.999, Q^2^ = 0.994). In female viscera, FA 18:1, FA 20:5, and FA 22:6 showed strong positive loading scores, whereas FA 20:1 and FA 22:1 showed strong negative loading scores. In male visceral tissues, several PC molecular species exhibited positive loadings, whereas FA 20:1 and FA 22:1 displayed negative loadings. Semi-quantitative levels of FA 20:1 and FA 22:1 were compared between the species for each sex in Figure 4E. FA 20:1 and FA 22:1, which are consistently elevated in capelin in both sexes, are promising species-specific lipid identification markers. Capelin primarily feeds on zooplankton [41], which inhabit the water off Iceland and Norway and are rich in C20:1 and C22:1 fatty acids [42], suggesting that dietary differences are the cause of lipidome variation. The lipid markers FA 20:1 and FA 22:1 identified in the viscera offer potential practical advantages for species discrimination. From the perspective of analysis time reduction, quantification of two FFA species promotes interspecies separation, simplifying the process compared to multivariate analysis required for other tissues, and contributing to determinations within tens of minutes. Furthermore, for food safety and traceability applications, this method supports the detection of imported capelin masquerading as shishamo, with the diet-derived FFA composition serving as a promising candidate marker suitable for simplified authentication during commercial distribution.

Overall, TAG enrichment in female shishamo heads and their compositional similarity with the roe suggest that the head serves as a TAG storage site and nutrient source for oogenesis. Meanwhile, high ω-3 PUFA concentrations in viscera reflect TAG hydrolysis-mediated release and reallocation, providing a biological basis for systemic energy supply networks. Additionally, FFAs generated during this visceral process may function as interspecific discriminant markers.

This study had several limitations. First, the reported lipid concentrations were semi-quantitative, as absolute quantification was not performed, and the values were derived relative to internal standards. Second, all specimens were sourced from commercial providers, introducing potential variability in handling, storage, and transport before analysis. Important biological and environmental factors that influence fish lipid composition, including diet, spawning stage, age, seasonality, and water temperature, have not been controlled for or systematically assessed. Additionally, post-harvest factors such as storage conditions and duration, particularly for imported capelin, may affect lipid stability, including the susceptibility of PUFAs to oxidation. Future studies should integrate controlled sampling, longitudinal monitoring of environmental and dietary influences, and absolute quantification approaches to validate and expand these findings.

## 4. Conclusions

This study used untargeted LC-MS to characterize the sex- and region-specific lipidomes of shishamo (*Spirinchus lanceolatus*) and capelin (*Mallotus villosus*). Female shishamo heads were enriched in glycerolipids, with a composition resembling roe, and uniquely contained very long-chain fatty acids (>C30) and ester-linked PCs. Both species exhibited elevated DHA and EPA levels in their viscera. Multivariate analysis highlighted clear region-specific lipid signatures; PCs predominated in head and meat, TAGs and CEs in the roe, and FFAs in the viscera. The lipid markers used for species discrimination were FA 20:1 and FA 22:1, which are abundant in capelin. Overall, these findings provide new insights into the nutritional lipid composition of shishamo and capelin, reveal region- and sex-specific lipid distributions, and identify lipidomic markers with potential applications in the authentication, nutrition, and tracing of geographic origin.

## Data Availability

The original contributions presented in this study are included in the article/Supplementary Materials. Further inquiries can be directed to the corresponding authors.

## Supplementary Materials

The following supporting information can be downloaded at: https://www.mdpi.com/article/10.3390/foods15020298/s1, Table S1: List of lipid metabolites and their relative amount (mg/100 g of body parts). Figure S1: OPLS-DA by sex and body part between shishamo and capelin, excluding viscera.
