# Supplementary figures and images for: Sex- and Regio-Specific Lipid Profiling of Shishamo and Capelin Fish by Nontargeted Liquid Chromatography/Mass Spectrometry
Source: Foods. 2026 Jan 14;15(2):298. doi: 10.3390/foods15020298 (PMC12840183; doi:10.3390/foods15020298)

## Female Head

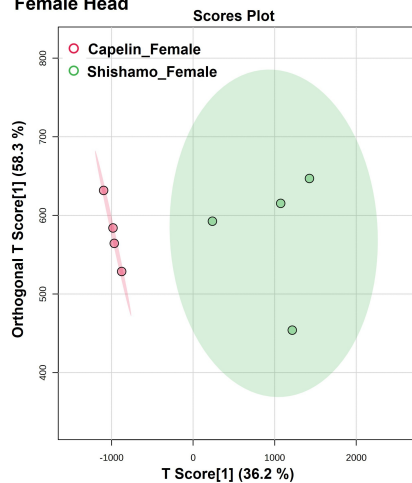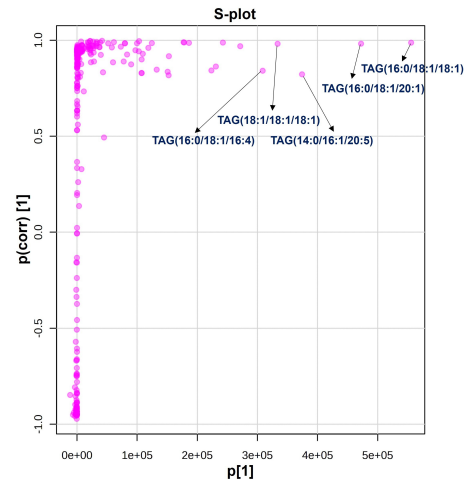

## Female Meat

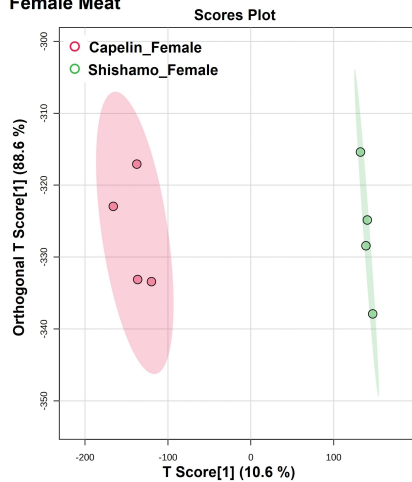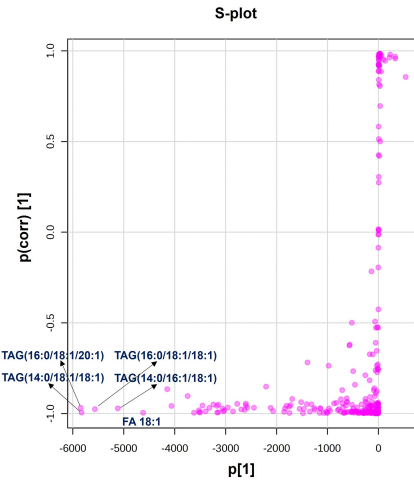

## Female Roe

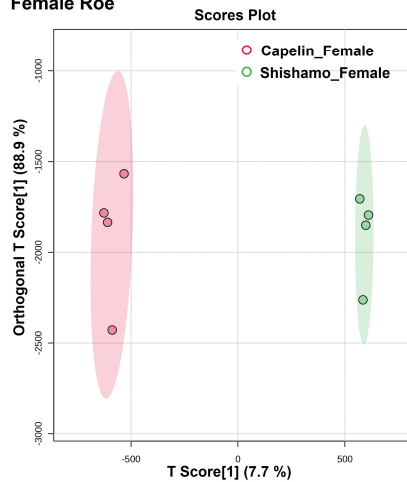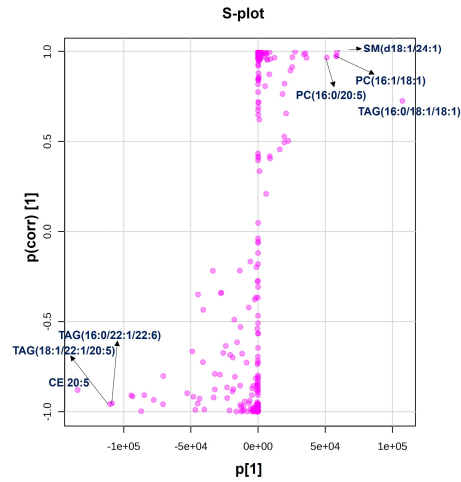

## Male Head

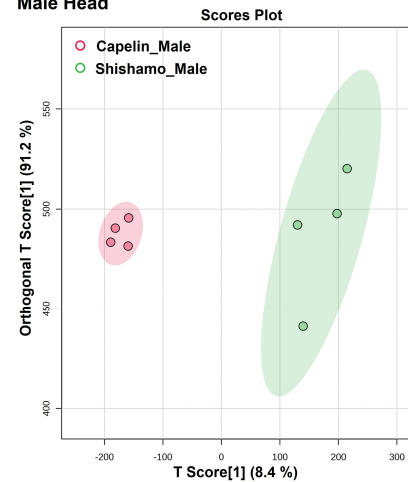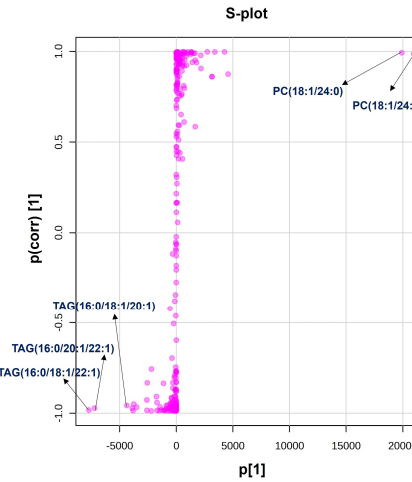

## Male Meat

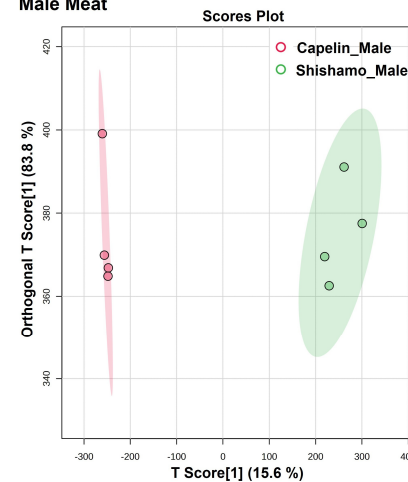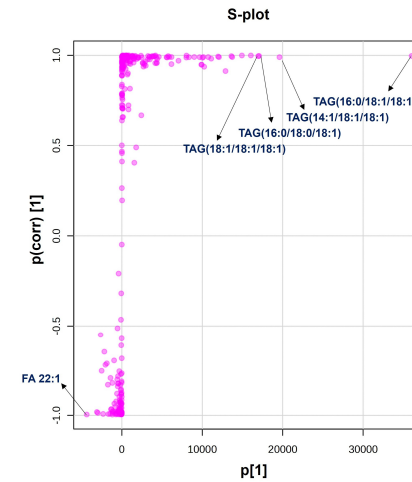

Supplement: Supplementary file 1 [file foods-15-00298-s001.zip › Supplementary Figure S1.pdf]
